# Soil bacterial communities shaped by geochemical factors and land use in a less-explored area, Tibetan Plateau

**DOI:** 10.1186/1471-2164-14-820

**Published:** 2013-11-22

**Authors:** Xiangyu Guan, Jinfeng Wang, Hui Zhao, Jianjun Wang, Ximing Luo, Fei Liu, Fangqing Zhao

**Affiliations:** Beijing Key Laboratory of Water Resources and Environmental Engineering, China University of Geosciences, Beijing, 100083 China; Beijing Institutes of Life Science, Chinese Academy of Sciences, Beijing, 100101 China; School of Ocean Sciences, China University of Geosciences, Beijing, 100083 China; State Key Laboratory of Lake Science and Environment, Nanjing Institute of Geography and Limnology, Chinese Academy of Sciences, Nanjing, 210008 China

**Keywords:** Bacterial community, Homogenization, Metagenomics, Soil, Tibetan Plateau

## Abstract

**Background:**

As the largest low-latitude permafrost region, the Tibetan Plateau (TP) is an important part of the earth’s terrestrial ecosystem and one of the most vulnerable areas to climate change and human activities. However, to the best of our knowledge, the bacterial communities in TP soils and their roles in biogeochemical cycles remain limited.

**Results:**

In this study, we report the bacterial community structure and function as well as their correlation with environmental factors in TP major ecosystems (farmland, alpine meadow and oligosaline lake) by using metagenomic approaches. Compared with other soil samples in various environments, TP soils share a core set of microorganisms with a distinct abundance and composition. Among TP soil samples, the taxonomic and functional composition of bacterial communities among the upper (3-5 cm) and lower (18-20 cm) soils of farmland sites were highly similar, whereas the dissimilarities within alpine meadow samples were significantly greater than among farmland samples. A similar pattern was observed in elements cycles and pathways associated with adaption to environment and land use types. Canonical correlation analysis revealed that the bacterial communities in most of farmland and alpine meadow soil samples were also significantly correlated with geogenic variables. Specifically, the root-nodule bacteria are negatively correlated with the soil moisture and pH, while *Thiobacillus* associated with sulfur cycles show potential responses to low temperature and intense UV radiation.

**Conclusions:**

These findings indicate that the bacterial community structure and functions in TP soils were influenced by both human activities and soil environmental properties, and that the bacterial communities appeared to be more homogenized in the farmland soils compared with pristine alpine meadows.

**Electronic supplementary material:**

The online version of this article (doi:10.1186/1471-2164-14-820) contains supplementary material, which is available to authorized users.

## Background

Soil is an essential ecosystem for organisms living on land. Soil microorganisms contribute to the breakdown of organic residues, element cycling and soil mineralization. Recent advances in high-throughput sequencing technologies have enabled comprehensive analysis of the microbial composition and function in a variety of soil environments, including farmland, forest, grassland, tundra, permafrost and desert [1–8]. These studies have revealed the high diversity of microbes in different niches and improved our understanding of new taxa, biogeographic distribution, and the association of specific microbial groups with geochemical factors.

The distribution of bacterial communities in different soil environments is controlled by a number of variables, including physical and chemical characteristics (e.g. depth, geogenic factors, pH, salinity and temperature), microenvironment structure (e.g. pore spaces, water films and dead organic matter), and other living organisms (e.g. arthropods, fungi). Geogenic factors and landforms have long been recognized as the drivers of soil geochemistry, but geogenic factors are fundamental determinants of microbial communities in naturally metal-rich soils in Australia [9, 10]. Nitrogen fertilization was shown to significantly influence bacterial community composition in a grassland soil and in agricultural soil in the United States while stimulating the distribution of Proteobacteria and Bacteroidetes [11]. The relative abundance of Acidobacteria, Actinobacteria, and Bacteroidetes changed with soil pH, which is believed to represent a prominent factor in bacterial community composition and proportion [6, 12]. The Tibetan Plateau (TP) has unique climatic and geochemical characteristics including high elevation (exceeding 4,500 meters on average), low temperature, limited nutrients and intense UV radiation [13–15]. Only a small number of studies have examined the microbial communities in glacier, snow, and lake sediments by sequencing of 16S rRNA in TP [16–19]. Consequently, the composition of microbial communities and the associated environmental factors in TP soils remain poorly explored.

Deep metagenomic analyses provide not only an understanding of microbial composition, but also comprehensive functional diversity of soil samples [1, 20]. Some integrated studies of microbial composition and function have provided insight into the relationship of taxonomic and functional structure and their different potential responses to environments across major global biomes such as permafrost thaw [21]. Composition and function of soil microbial communities from cold deserts, hot deserts, forests, grasslands and tundra have been compared, and some functional genes associated with osmoregulation, dormancy and antibiotic resistance have been shown to be related to environments [20]. Additionally, soil samples from different sampling times and depths in the same grassland and their bacterial structure and functional fluctuations have been sequenced and analyzed [1]. Metagenomic approaches can be used to explain how particular pathways reflect the adaptation of microbial communities to environments. For example, the abundance of genes for amino acid synthesis, vitamin and cofactor metabolism, and lipid and glycan metabolism can indicate bacterial community adaptation to specific environmental challenges, nutrient-limited conditions and physicochemical conditions respectively [22].

As the third pole of the world, although TP is still in a relatively pristine state, pristine alpine meadows have been converted to farmlands in recent years [13–15]. We collected 20 soil samples from ten different locations to cover the major ecosystems of alpine meadow, farmland and lake in the TP. Among these ecosystems, alpine meadow soil was found to have significantly higher organic carbon content than soils from other types of ecosystems [14, 23, 24], which account for more than 60% of the TP [25–27]. Farmland is the major ecosystem affected by human activity in this region [28]. Namco Lake (oligosaline water), which is one of the three largest lakes in TP, has shown increasing water levels owing to glacial melt [29, 30]. Previous studies have shown that bacterial communities and functions were not only affected by environmental heterogeneity, but can also be influenced by the strength of human activities and land use [11, 31, 32]. Moreover, the top 10 cm of soil layer was found to be more easily influenced and variable than the lower soil layer [33]. Therefore, in this study, samples were collected from the upper (3-5 cm) and lower (18-20 cm) soil layers for assessment of bacterial community diversity and anthropogenic impact on them. Additionally, we compared whether the degree of similarity in bacterial communities or functions between surface and lower layers in farmlands differed from those in pristine alpine meadows. To accomplish this, we measured a number of physical and chemical properties of the TP soil samples and then performed metagenomic sequencing of the total DNA extracted from them. We then integrated this data to identify taxonomic and functional diversities of microbial communities in TP soils and to assess potential environmental factors and land use that may shape the bacterial communities.

## Methods

### Sample collection and soil analyses

**Figure 1:**
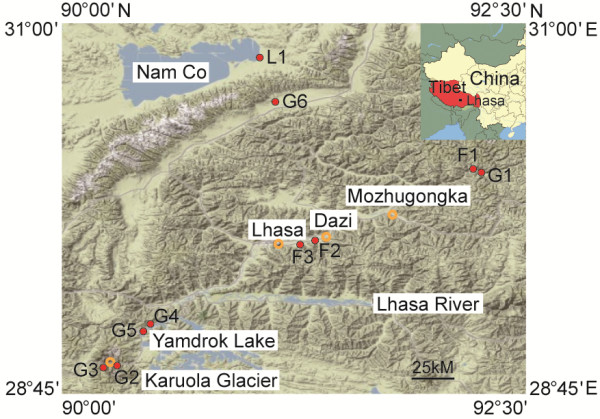
**Sampling sites.** Geographic locations of 10 sampling sites. F1-3 samples are from highland barley farmland in which F1 is far from residential area and F2 and F3 are near to residential area; G1-6 samples are from alpine meadows; L1 sample is from Namco Lake.

Twenty soil samples were collected at ten different sites (Figure 1) from TP in September 2011, including alpine meadow sites (G1-6 with less anthropogenic activities), farmland sites (one far from residential area F1 and two near residential area F2 and F3, conversed from alpine meadow sites for decade), and one site in water and land intersection of Namco Lake L1. At each sampling site, we collected both the upper (3-5 cm) and the lower (18-20 cm) layers of the soil, and triplicate samples were taken at the vertex of triangle with edge distance of 1 m. Hereafter, we use F/G/L to represent three land types of farmland, grassland and lake, respectively, and use U/L to represent the upper and lower soil layers, respectively. Soil samples were collected using stainless steel sample tubes (diameter 40 × 40 mm, length 22 cm), and these tubes were immediately put into an incubator with ice packages. The soil samples at 3-5 cm and 18-20 cm layers from triplicate sampling tubes were taken and mixed under a sterile condition as soon as possible after we returned to the lab, and then divided into two parts stored at 4°C for chemical test and -20°C for DNA extraction within 12 h, respectively.

Soil pH value was determined in a soil/water (1:5 w/v) suspension by using pH meter (Sartorius PB-10, Goettingen, Germany). Total organic carbon (TOC) was determined using the K_2_Cr_2_O_7_ oxidation method [34], and total nitrogen (TN) was analyzed by Eurovector elemental analyser (Isoprime-EuroEA 3000, Milan, Italy). Total sulfur (TS) was measured by an infrared absorption method after high frequency combustion (High-speed analyzer HWF-900A, Jinan, China). Other trace metals were analyzed by ICP-MS (Thermo Fisher X-series, Franklin, MA, USA) and ICP-AES (TJA IRIS-Advantage, Franklin, MA, USA). Analysis on volatile organic compounds (VOCs) of soil samples was performed with an automatic static headspace sampler. The samples were analyzed by a GC/MSD System (Agilent 6890/5973 N, Palo Alto, CA, USA). The reporting limits were 1.0 μg/L for all the analytes.

### DNA extraction and metagenomic sequencing

DNA of homogenized samples (0.5 g) was extracted by the PowerSoil DNA extraction kit (MoBio Laboratories, Carlsbad, CA, USA), and stored at -20°C for further use and -80°C for permanent preservation. The quantity and quality of isolated DNA were evaluated using a Nano Drop spectrophotometer (Thermo Fisher Scientific ND-1000, Waltham, MA, USA) and agarose gel electrophoresis (Bio-Rad, Hercules, CA, USA), respectively. For each sample, 0.5 μg of purified metagenomic DNA was sheared into fragments of ~180 bp in length, and a library was constructed according to a standard protocol provided by Illumina, Inc. (San Diego, CA, USA). Quantification was performed using a Qubit Fluorometer (Invitrogen, Life Technologies, Grand Island, NY, USA) and a Stratagene Real-time PCR Cycler (Agilent Mx3000P, Santa Clara, CA, USA) prior to cluster generation in a c-Bot automated sequencing system (Illumina, Inc., San Diego, CA, USA). Eight libraries with different indexes were pooled together and sequenced in one lane using an Illumina HiSeq 2000 high-throughput sequencing instrument with 2 × 100 bp paired-end (PE) sequencing. A total of two and a half lanes were sequenced for the 20 libraries.

### Quality filtering and PE reads merging

Firstly, low quality PE reads were removed before further analysis by custom scripts. Secondly, quality-filtered reads were merged based on the overlap of PE reads. Briefly, we iteratively aligned a pair of reads (read 1 and read 2) with an overlap length ranging from 6 to 40 bp. In each iteration, the overlap score was calculated as the number of mismatches divided by the overlap length. If the score of the best overlap was smaller than the mismatch threshold (0.15), read 1 and read 2 were merged into a long read. The WGS sequencing data were deposited in the Sequence Read Archive (SRA) database with an accession number as SRP032429.

### Metagenomic analyses

Sequences were aligned and annotated using the Basic Local Alignment Search Tool (BLASTX) algorithm and the National Center for Biotechnology Information (NCBI) non-redundant (NR) sequence database with an E-score cutoff value of <10^-4^, and the BLASTX hits were further processed by the Metagenome Analyzer program (MEGAN, V4) to statistically analyze the abundance of taxa in each sample [35]. After normalizing sequence counts of each taxonomic group by the total number of reads, statistical analysis was performed on the bacterial composition and abundance at the phylum and genus levels. To compare the taxonomic communities between TP and other soil environments, metagenomic datasets were obtained from MG-RAST [36], and similar annotation analyses were carried out as below.

Metagenomic reads were assembled using SOAPdenovo2 v2.04 [37], and certain assembled contigs were further scaffolded using PGA [38]. The MetaGeneMark v2.8 gene prediction tool [39] was used to predict genes from assembled contigs. The predicted proteins were compared against the NR database using BLASTP with an E-score cutoff value of <10^-2^ and were taxonomically and functionally annotated using a combination of lowest common ancestor and consensus approaches [40]. Metagenomic sequences were functionally annotated using the Kyoto Encyclopedia of Genes and Genomes (KEGG) bioinformatics database [41] and MetaCV [42]. Functional categories and genes in the KEGG pathway were counted in each sample for further functional analysis. Certain reads were extracted from KEGG sulfur metabolism pathway and BLASTXed against the NCBI NR protein database with an E-score cutoff value of <10^-2^. The alignment results were parsed by MEGAN to determine the abundance of each bacterium in contributing to sulfur metabolism. Complete genome of *Thiobacillus denitrificans* ATCC 25259 (NC_007404.1, 2.91 Mb) was chosen as a reference to calculate the sequence frequency of each genotype of *Thiobacillus* reads from samples of G5U, and Burrows-Wheeler Aligner (BWA) [43], SAMtools [44] and the Integrated Next-gen Genome Analysis Platform (inGAP) [45, 46] were used as mapping, calculating and visualization tools, respectively.

### Statistical analyses and visualization

Alpha diversity was calculated by using the taxonomic and functional metrics (Simpson Diversity Index, 1/D). The dissimilarity in taxonomic and functional composition between samples was measured with Bray-Curtis distance [47]. The variation of dissimilarity was compared between farm and meadow ecosystems by focusing on different sample groups, that is the upper, the lower and the lower vs. upper samples. The significance between the sample groups was tested using *t*-test with permutations (999). All the analyses were carried out in R.

Principal component analysis (PCA) implemented in imDEV [48] was performed to evaluate the similarity among the twenty metagenomic communities from TP based on the metadata of taxonomic composition, functional annotation and environmental factors. We further use Canonical Correlation Analysis (CCA) to measure the relationship between environmental factors and sampling sites in CANOCO 4.5 (Biometrics Wageningen, The Netherlands). The number of genera and functional reads were counted using the annotation results from MEGAN.

## Results

### Geochemical measurements of 20 soil samples

Geochemical and field data for the sampling sites are shown in Additional file 1: Table S1 and S2. TN was the highest in alpine meadow G2, TS was significantly higher in alpine meadow samples G4U/L and G5U/L than all other samples, and G2 and G/L4 had higher TOC than other studied samples. The C: N and C: S ratios are important measures of geochemical cycling, and the C: N ratio can strongly influence the bacterial community structure [4, 49]. The C: N ratio in samples L1U/L, G5U/L, and G3U was more than 30:1. In samples G3L, G6U, and G4U/L, the C: N ratio was between 20 and 30, and nitrogen was neither fixed nor mineralized. The C: N ratio in the remaining samples was lower than 20, indicating that nitrogen was likely mineralized. The C: S ratio was below 200 in all samples, indicating sulfur mineralization. The phosphorous content of the samples was ranked as follows: farmland (~0.1%) > alpine meadow (<0.1%) > saline lake (<0.001%). The K: N and K: P ratios varied among soil samples, but all were more than 14 and 134, respectively, indicating they were rich in potassium. The VOCs were below the limit of detection in all samples.

### Bacterial community composition and diversity in TP major ecosystems

Sequencing of both the upper (3-5 cm) and lower (18-20 cm) soil samples from 10 sampling sites in TP resulted in a total of 336 million read pairs. The paired reads were merged into 61 million long sequences (160-197 bp each), and these PE-merged sequences were compared against the NCBI NR protein database by BLASTX. In different samples, 48.2-58.3% of these PE-merged sequences could be assigned to eubacteria, 0.8-3.3% to eukarya, ~0.1% to both archaea and viruses, while the remaining reads could not be assigned.

**Figure 2:**
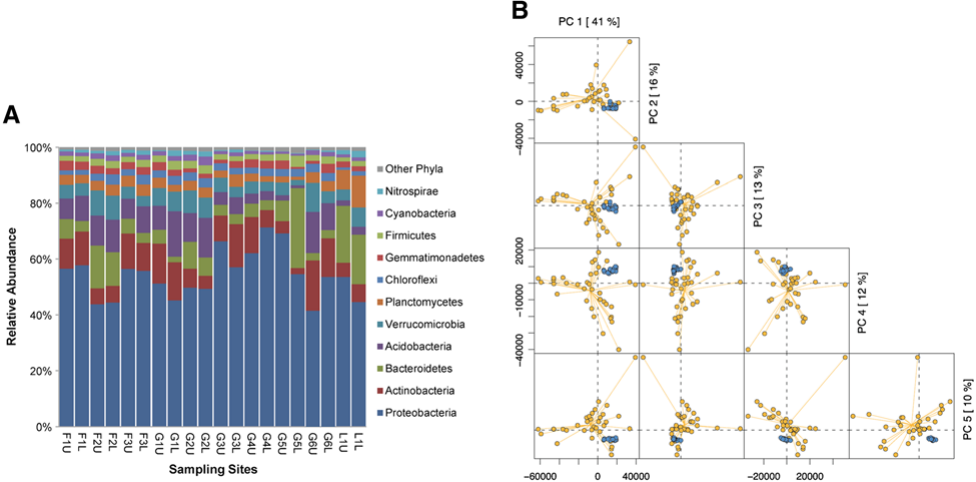
**Bacterial phyla and clustering for the soil samples from the Tibetan plateau (TP). (A)** Relative abundance of the identified bacterial phyla in the 20 soil samples. Detailed data of each phylum are listed in Additional file 1: Table S3. **(B)** Principal component analysis (PCA) of various soil samples based on the composition and abundance of their bacterial communities at the phylum level. Blue dots represent the TP samples in this study. Yellow dots represent previously reported soil samples from various environments.

To examine the bacterial communities in greater details, the MEGAN analysis pipeline was used to parse BLAST hits and to estimate bacterial species distribution and abundance. Proteobacteria was the most abundant phylum (41.4-71.2%) in every sampling site, followed by Actinobacteria, Bacteroidetes, Acidobacteria, and Verrucomicrobia (Figure 2A). These five phyla accounted for more than 78.4% of the total bacterial community in each sample. However, the rank order of the most abundant phyla varied among samples in Namco Lake samples, Bacteroidetes (17.8-20.4%) was more abundant than Actinobacteria (5.1-6.4%) (Additional file 1: Table S3).

**Figure 3:**
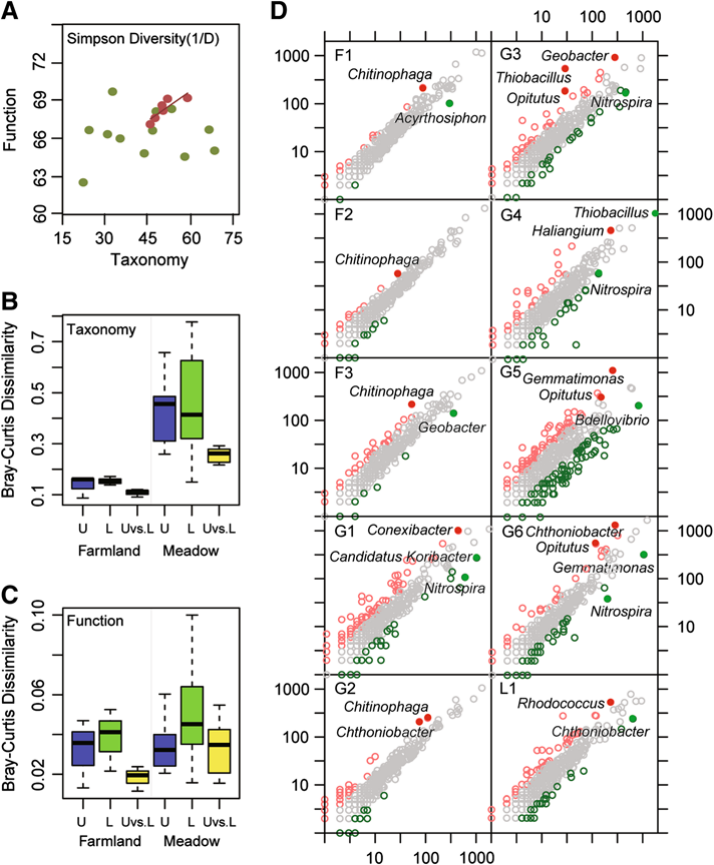
**Comparisons of bacterial communities taxonomic and functional diversities between farmland and alpine meadow soils in TP. (A)** Relationships between bacterial taxonomic and functional alpha diversity (Simpson Diversity Index, 1/D) of farmland samples (red dot) and alpine meadow samples (green dot). **(B)** Box plot of the Bray-Curtis distances estimated based on taxonomic composition of bacterial communities in upper and lower layers of TP soils. U: Upper (blue), L: lower (green) and U vs. L: upper vs. lower (yellow) layer soil samples from two major TP ecosystems farmlands (left) and alpine meadow (right). Components of the box are: top of the box, upper hinge; midline of box, median; bottom of box, lower hinge; bars, 1.5 times length of box (1.5 times the horizontal spread); dots, values that are larger or smaller than 1.5 times the horizontal spread of the distribution, plus the upper or lower hinge. **(C)** Box plot of the Bray-Curtis distances estimated based on functional composition of bacterial communities in upper and lower layers of TP soils. **(D)** Comparison of the bacterial richness between the upper (3 ~ 5 cm) and the lower (18 ~ 20 cm) layers of the soil samples at 10 different sites. The red circles represent the genera which have at least twice the amount of bacteria in the upper layer than in the lower layer; the green circles represent the genera which have at least twice the amount of bacteria in the lower layer than in the upper layer.

Alpha diversities of Namco lake samples were relatively lower than those among farmland samples and alpine meadow soil samples (Additional file 1: Table S4). The alpha diversity of bacterial composition and function of farmland samples were similar, and their taxonomic alpha diversity was correlated with functional alpha diversity (R^2^ = 0.7234) (Figure 3A). The alpha diversities of bacterial communities were more variable among alpine meadow and Namco lake samples than farmlands samples, and the correlation between their taxonomic and functional alpha diversity was not obvious (Figure 2B). There was no correlation between taxonomic and functional beta diversity among TP samples.

**Figure 4:**
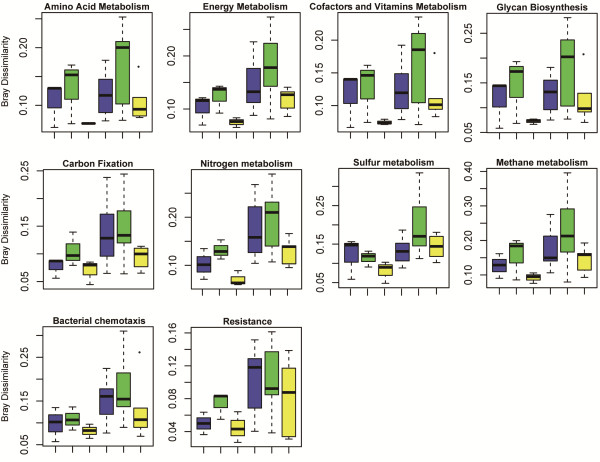
**The dissimilarity of several functional composition between upper (blue), lower (green) and upper vs. lower (yellow) layer soil samples from two major TP ecosystems farmlands (left three boxes) and alpine meadow (right three boxes).** Components of the box are: top of the box, upper hinge; midline of box, median; bottom of box, lower hinge; bars, 1.5 times length of box (1.5 times the horizontal spread); dots, values that are > or <1.5 times the horizontal spread of the distribution, plus the upper or lower hinge. The variation of dissimilarity was compared between farmland and alpine meadow ecosystems computed with Bray-Curtis based on relative abundance of functional genes in the KEGG pathway.

Bacterial taxonomic and functional dissimilarities (Bray-Curtis distances) were computed among upper, lower and upper vs. lower layer samples from farmlands and alpine meadows and then used for statistical analysis. Bacterial taxonomic dissimilarities among upper or lower samples from different alpine meadow sites were significantly (P < 0.05) higher than those of farmlands (Figure 3B, P values were shown in Additional file 1: Table S5). No significant difference in bacterial composition was observed between upper and lower layer samples from among farmlands, and the dissimilarities among alpine meadows were (P < 0.05) higher than farmlands (Figure 3B). Evaluation of the functional composition revealed that upper layers of alpine meadows and farmlands exhibited a similar degree of dissimilarity, whereas alpine meadows were more divergent than farmlands (Figure 3C). Similarly, alpine meadow samples (especially lower layer samples) showed significantly (P < 0.05) higher dissimilarities than farmlands in metabolic pathways of amino acid synthesis, energy metabolism, vitamin/cofactor metabolism, and lipid/glycan metabolism, which were involved in the bacterial community adaptation to specific environmental and nutritional conditions (Figure 4A-D). Likewise, dissimilarities of upper lower and upper vs. lower samples from alpine meadows were all significantly (P < 0.05) higher than those of farmlands in carbon, nitrogen, sulfur, and methane metabolism cycles (Figure 4E-H). For bacterial chemotaxis and resistance to antibiotics and toxic compounds pathways associated with human activities, the dissimilarities are also significantly higher in alpine meadow than in farmlands. Furthermore, the functional dissimilarities between upper and lower samples in antibiotics and toxic compounds from alpine meadows were significantly (P < 0.05) higher than those from farmlands (Figure 4I-J).

### New bacterial resources and similar bacterial core

To investigate the taxonomic diversity among samples, the similarity between assigned metagenomic reads and their reference sequences were compared and analyzed (Additional file 2: Figure S1). We found that the vast majority of reads shared 65-85% sequence identity with known species. One genus, *Thiobacillus*, had higher sequence identity with soil metagenomic sequences. The majority of metagenomic sequences classified as *Thiobacillus* had ≥ 90% identity at the amino acid level with known sequences from *T. denitrificans* ATCC 25259, which is a facultatively anaerobic, chemolithotrophic, sulfur-oxidizing bacterium with an ability to couple the oxidation of inorganic sulfur to the reduction of oxidized nitrogen compounds (such as nitrate, nitrite) to dinitrogen. Interestingly, we found that a small fraction of reads in F1 could be assigned to an insect, *Acyrthosiphon pisum*, with 90-95% sequence identity.

The distribution of the top 50 most abundant genera revealed a core set of taxa shared by the 20 samples and was different from other environmental samples such as tundra, grasslands, farms, forests, lakes, creeks and marine systems (Additional file 3: Figure S2). The core microbiota of TP samples included *Candidatus Solibacter*, *Gemmatimonas*, *Sorangium*, *Nitrospira*, *Chthoniobacter* and others. Samples from farmlands and alpine meadows shared a more similar core of genera than other environmental samples. It should be noted that samples from alpine (swamp) meadow G4U/L and its adjacent site G5U/L had the largest fraction of *Thiobacillus*. The bacterial composition and abundance of saline lake samples L1U/L were obviously different from other TP samples. The proportion of core genera in L1U/L differed from other TP samples in that it contained *Thiobacillus*, *Conexibacter*, *Nitrospira*, *Burkholderia*, *Chitinophaga*, and a few others.

Comparison of the bacterial communities between upper and lower layers of TP soils is shown (Figure 3D). Most of genera showed an unobvious diversity (grey color), and the difference between upper and lower layers samples were mostly low abundance genera (less 0.1%). The bacterial communities in both layers of F1, F2, and F3 were very similar, and a slight difference was only three microorganisms including *Chitinophaga*, *Geobacter* and *Acyrthosiphon*. Proglacial alpine meadow G2 was lowest diversity among alpine meadow samples, in which *Nitrospira* as nitrifying bacteria was the common genus at more than twice the amount of bacteria in the lower layer than in the upper layer except G2. Upper and lower layer samples from G5 were the more divergent than other sites samples, and the differences were mainly from genera including *Thiobacillus*, *Geobacter*, *Gemmatimonas*, *Opitutus* and so on. These observations showed that the bacterial communities at depth of 3-5 and 18-20 cm in soil from various farmland and alpine meadow soil environments were really similar with minor differences in root-associated bacteria and nitrifying bacteria (more abundant in upper soil than lower soil) and denitrifying bacteria (less abundant in upper than lower soil).

### PCA and CCA based on metagenomic data

**Figure 5:**
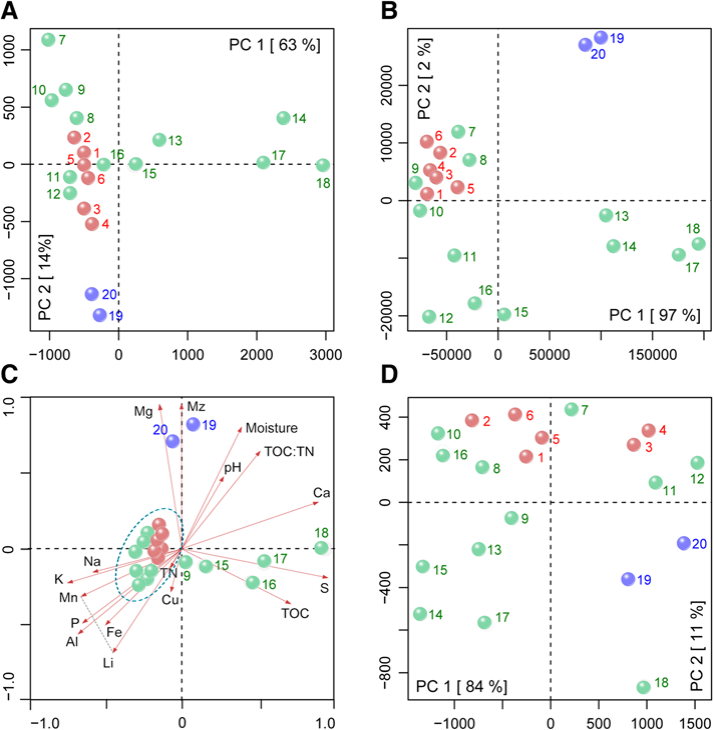
**Statistical analyses of the 20 metagenomes and their associated environmental factors.** Colored spheres represent the TP soil samples: red, farmlands (sites F); green, alpine meadow (sites G); blue, lake (site L). Sample names are abbreviated due to space constraints: 1, F1U; 2, F1L; 3, F2U; 4, F2L; 5, F3U; 6, F3L; 7, G6U; 8, G6L; 9, G1U; 10, G1L; 11, G2U; 12, G2L; 13, G4U; 14, G4L; 15, G3U; 16, G3L; 17, G5U; 18, G5L; 19, L1U; 20, L1L. **(A)** PCA based on the relative abundance of all bacterial genera. **(B)** PCA based on soil environmental factors. Detail data of these factors was deposited in Additional file 1: Table S1 and Additional file 1: Table S2. **(C)** CCA based on normalized soil environmental factors and the relative abundance of all bacterial genera. MZ means grain size; Triangular region formed by red arrows of Mn and Li, and the gray line between them indicates the range of minor elements’ distribution; Samples (1-8, 10-14) are densely clustered, and their names are not shown. **(D)** PCA based on relative abundance of functional genes at the third KEGG pathway level.

To elucidate the similarity of the bacterial community composition and abundance between TP and other soil environments, we downloaded the taxonomic composition of the 24 different metagenomes from MG-RAST (Additional file 4: Table S6). PCA was performed based on normalized phylum counts. The first five principal components (PC1 41%, PC2 16%, PC3 13%, PC4 12% and PC5 10%) explained 92% of the total variance (Figure 2B). Although the phyla were not fundamentally different from other biomes, we found that the bacterial communities in the 20 TP samples were more similar to each other than to bacterial communities from any other environmental samples. To investigate the variation among TP samples, we further performed PCA based on the taxonomic composition (Figure 5A). Samples from farmland sites (F1-3) were grouped together along PC1 (63%). Alpine meadow and lake samples were dispersed away from this group, and upper and lower layer samples from most of these sites were separated along PC1. Samples (L1, G4 and G5) with high moisture (>30%) were more divergent on soil samples from different layers. Upper and lower layer samples from G2 with both high TOC and TN were not divergent and mixed together with farmland.

In addition to bacterial community composition, we also performed PCA of both environmental factors and functional annotations. Functional annotations were assigned to metagenomic sequences by a TBLASTX comparison to the non-redundant KEGG database. In contrast to the PCA of bacterial community composition, samples from sites G1 and G6 were clustered within the farmlands, and G2 was separated from the cluster of farmland samples, mainly correlated with moisture, soil carbon, nitrogen, and sulfur concentrations (Figure 5B). The sites of bacterial community functional profiles showed the greatest dispersion among the three PCA (Figure 5D). Although functional profiles of samples from F1-3 showed a closer relationship than samples from farmlands and lake, they could not be distinguished completely along PC1 (84%) (Figure 5D).

CCA was performed to explore the relationship of environmental factors and bacterial community composition. The results revealed a strong association between moisture, magnesium, calcium and saline water-saturated samples (L1U/L) (Figure 5C). G4 and G5 were clearly controlled by the TS and TOC concentration, while the other samples from farmland and meadow sites were clearly correlated with all minor elements and lithophile major elements except magnesium and calcium.

### Nitrogen and sulfur-associated bacteria

**Figure 6:**
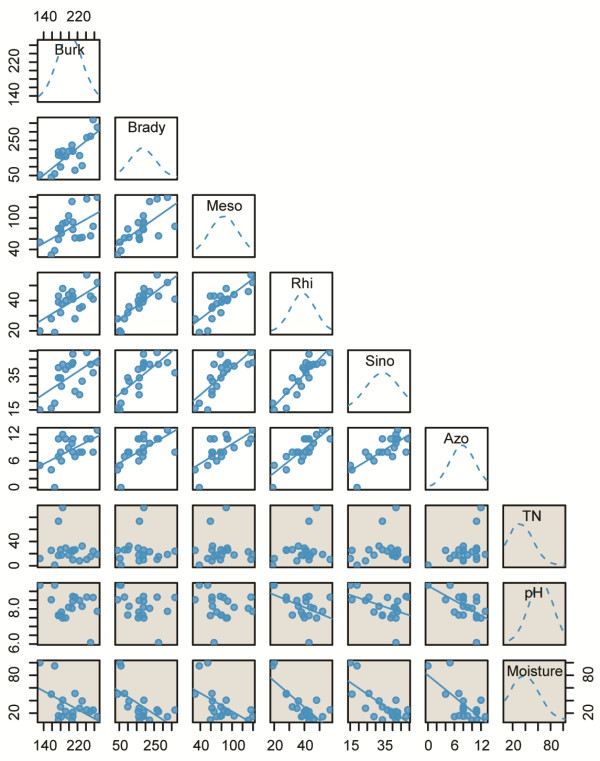
**Correlation between root nodule bacteria and total nitrogen (TN), pH value and moisture in TP soil samples.** Burk, *Burkholderia*; Brady, *Bradyrhizobium*; Meso, *Mesorhizobium*; Rhi, *Rhizobium*; Sino, *Sinorhizobium*; Azo, *Azorhizobium*. Blue curves show normal distribution of values in each sample. Pearson index was used to evaluate the level of correlation. Backslash lines indicate negative correlation (r = 0.45 ~ 0.79, P < 0.05).

As shown in Figure 6, the abundance of the root-nodule bacteria *Burkholderia*, *Bradyrhizobium*, *Mesorhizobium*, *Rhizobium*, *Sinorhizobium* and *Azorhizobium* was positively correlated with each other across different TP soil samples. Interestingly, all six of these genera were negatively correlated with the moisture levels (Pearson rank correlation test: r = 0.51-0.79, P < 0.01), while no obvious correlation with TN. Among α-proteobacteria, the abundance of *Rhizobium*, *Sinorhizobium* and *Azorhizobium* was negatively correlated with pH values (Pearson rank correlation test: r = 0.45-0.59, P < 0.05) (Figure 6).

Reads assigned to *Thiobacillus* in G5U were aligned to the complete genome sequence of *T. denitrificans* ATCC 25259, which revealed an uneven distribution of the depth of coverage (Additional file 5: Figure S3). High frequency genes included sulfur metabolism-related genes, the NADH dehydrogenase subunit, a heavy metal-related gene and a cold-shock DNA-binding protein family, whereas nitric oxide reductase occurred at a low frequency (Additional file 4: Table S6).

## Discussion

TP is the highest and largest plateau on earth and is considered one of the most fragile ecosystems [25]. However, only a very limited number of studies have explored the microbial communities in TP soils by using 16 s rRNA gene clone library approaches to date [16, 50]. Therefore, in this study, we generated and analyzed 67.2 Gb metagenomic sequences from 20 soil samples to assess and quantify the variation in bacterial communities between major ecosystems in TP soils.

Comparison of the TP bacterial communities with other publicly available metagenomes (MG-RAST) showed that the Tibetan samples formed a distinct cluster (Figure 2B). The TP soil communities were distinct despite the fact that the soils share some physicochemical characteristics and land-use with soils from other environments such as arctic areas or farmland. Although phyla are detected in all compiled soils (including TP), the relative frequencies of bacterial phyla vary in different niches, which are generally thought to be controlled by local environmental variables including thawing, nitrogen gradients, pH gradients and other geochemical factors [11, 51]. TP has created a separate group from other environmental samples with similar phyla but different abundance (Figure 2A). Proteobacteria was the dominant phylum in high pH soil from shrub-covered permafrost and farmland [2]. In metal-rich soils with low carbon and nitrogen, some genera of *Alpha*-*proteobacteria*, which are capable of nitrogen fixation, are present in high levels [9]. In tundra soil, Actinobacteria was the dominant phylum due to its capacity to maintain metabolism and DNA repair at a low temperature and to degrade complex organic matter in soil [5, 52]. These observations are consistent with our findings in TP soils owing to the low-nitrogen and low-temperature of soils in these regions [5].

Soil samples from the TP shared a similar core microbiota compared with other environmental samples (Additional file 3: Figure S2). The core microbiota in samples from L1 differed from other TP samples, but the most abundant were relatively similar across all TP soil samples when compared with other environmental water, soil and sediment samples. These predominant genera are involved in nutrient element supplies, and phosphorus, sulfur, nitrogen cycles and these soil bacteria depend on and interact with each other while metabolizing the many raw materials present in soil. *Gemmatimonas* is a polyphosphate-accumulating genus for plant absorbing essential phosphorus in nutrient deficient TP areas [53]. *Thiobacillus* has the ability to couple denitrification to sulfur-compound oxidation [54]. Genera participating in the nitrogen cycle were also abundant in TP soil. *Nitrospira*, a nitrite-oxidizing bacteria, converts nitrite to nitrate as central step in the nitrogen cycle [55]. *Chthoniobacte*r is involved in the transformation of organic carbon compounds in the soil, but does not have the capacity to fix nitrogen [56]. *Anaeromyxobacter* can reduce nitrate and nitrite to ammonia, and nitrous oxide to nitrogen gas under both oxic and anoxic redox conditions [57]. *Opitutus* species can produce acetate and propionate and hydrogen, which are important in methanogenesis, and can reduce nitrate to nitrite [58]. These nitrogen cycle associated genera also obviously differed in abundance in upper and lower layer soil samples.

Alpha diversity of the bacterial communities from Namco Lake was lower than all farmland and meadow sites in TP due to the oligotrophic environment of lake. Farmlands investigated in this study had been converted from alpine meadows within a decade. Although anthropogenic disturbance of farmlands did not increase taxonomic alpha diversity, taxonomic beta diversity decreased significantly relative to alpine meadows (Figure 3A and 3B). A similar pattern has been reported in other studies of microbial communities in Netherlands pine forest soils and Amazon agriculture soils [4, 59]. It is worth noting that functional dissimilarities of alpine meadow sites were also higher than those of farmlands in both upper and lower layers of soil samples (Figure 3C). The metabolic pathways involved in amino acid synthesis, energy metabolism, vitamin and cofactor metabolism and lipid and glycan metabolism were considered to be responsible for bacterial adaptation to special environmental conditions [22]. We found that the dissimilarities of these pathways in farmlands were lower than those of alpine meadows, indicating that human activities might drive the homogenization of bacterial communities and their functional gene pools in TP farmland soils (Figure 4). Similarly, dissimilarities in elemental metabolism cycles such as carbon, nitrogen, sulfur and methane among alpine meadows were also higher than those among farmlands, which further confirmed that the bacterial communities in alpine meadows were more diverse with respect to elements utilization and energy conversion without human disturbance. Loss of diversity with persistent human disturbance will increase the risk of abrupt and potentially irreversible ecosystem collapse [60]. Homogenization of soil bacterial communities of farmlands converted from alpine meadow is also likely to make TP ecosystems vulnerable to environmental changes.

Differences between bacterial communities at depth of 3-5 and 18-20 cm in TP soil were mostly observed among root-associated bacteria and nitrifying bacteria (more abundant in upper soil than lower soil) and denitrifying bacteria (less abundant in upper than lower soil) (Figure 3D). Nitrifying bacteria prefer aerobic conditions, whereas denitrification is likely to occur in anaerobic environments [61]. Some genera are indicators of different environmental factors. For example, *Haliangium* species are obligate halophiles [62] that differed in abundance between upper and lower layers of the alpine meadow G4. These genera varied between layers owing to variations in soil oxygen and pH. Taken together, these results highlight that human disturbance homogenized the bacterial communities in both composition and function at depths above 20 cm.

Bacterial community composition in TP samples was influenced by a number of geochemical factors including carbon, nitrogen, sulfur, temperature and moisture. In all PCA based on environmental factors, taxonomic composition, and functional composition, samples from three farmland sites (far or near residential area) were clustered together, and all other samples were dispersed, that they varied greatly (Figure 5A-C). CCA plots of sampling sites and environmental factors suggested that TS may control the taxonomic composition of G4 and G5 samples, while saline water saturation may affect L1 samples (Figure 5D). All remaining samples were positively correlated with most of the major and minor elements. Changes in pH value in the studied soil samples were not relevant to taxonomic composition. Viles [10] reported that vegetation changes, land use, and anthropogenic activities influenced soil microbial communities. Soil samples collected from four regions of Australia showed that their microbial community composition was primarily affected by geochemistry and secondarily by other factors [9]. The bacterial community composition of F1 (far from residential area) and F2/3 (near residential area with fertilization) were nearly the same, indicating that these geochemical factors and land use both shaped bacterial communities.

Evaluation of the main element cycle and functions of bacterial communities revealed that the availability of nitrogen and sulfur and moisture of samples influenced the composition of different TP bacterial communities. Nitrogen fixation is very important for plant growth in the TP. Abundant bacteria in the Tibetan samples included *Burkholderia*, a *β-proteobacteria* genus associated with root-nodules. Root-nodules bacteria are capable of establishing a nitrogen-fixing symbiosis on legumes or other host plant roots [63]. The moisture differences among the twenty Tibetan samples were negatively correlated with five Rhizobia genera (Figure 6). The abundance of *Bradyrhizobium*, *Mesorhizobium*, *Rhizobium* and *Sinorhizobium* influenced by moisture was also found in the north China Plain, but the relationship between Rhizobia and pH values differed between them [6, 64]. Due to climate warming and glacial melting, deposition of reactive nitrogen in soils is increasing [65]. Accordingly, the impact of such environmental changes on soil microbial communities should be further investigated in alpine meadows associated with glaciers.

The sulfur cycle is another important biogeochemical cycle, because sulfur is a constituent of many proteins and cofactors [66]. *Thiobacillus* was the dominant genus in alpine meadow G4 and G5, which had the highest sulfur compared to other samples. *Thiobacillus* is a chemolithoautotrophic sulfur-oxidizing *β-Proteobacteria* capable of nitrate-dependent Fe (II) and U (IV) oxidation [54, 67, 68]. The different number of reads assigned to various *T. denitrificans* protein-coding genes further reflected its genomic potential adaption to local environmental factors. The elevated sequencing depth coverage in sulfite reductase genes and cold-shock genes reflect the adaption to a high sulfur concentration and low temperature (Figure 6 and Additional file 4: Table S7). A higher frequency of NADH dehydrogenase genes, which catalyze the transfer of electrons from NADH to coenzyme Q, contributes to UV radiation resistance [69, 70] (Additional file 5: Figure 3).

This study presented the first comprehensive assessment of bacterial community structure and function in TP soils. However, considering the complexity of soil microbial community and the less exploration of TP soils, there were still a lot of difficulties in decoding these metagenomic data. The microbial community in soils especially in pristine environments is dominated by novel microorganisms, which are lack of closely related reference sequences in known public genomic databases. Homology based approaches, such as NR-BLAST method plus the lowest common ancestor (LCA) algorithm, tend to classify metagenomic sequences into higher taxonomic levels. A similar problem also occurs in functional annotation, which may lead to inaccurate annotation or the absence of some important and key functional genes. This is a universal problem that puzzles all metagenomic studies in microbial ecology. As the number of sequenced species grows, the situation will be improved.

## Conclusions

TP soil bacterial community structure and functional profiles exhibit several distinct features from other environments, which are associated with the adaptation to environmental factors and land use. The beta diversity of bacterial communities in farmland decreased due to anthropogenic activities by homogenization even at different soil depths. As a much less explored environment, the metagenomic sequences and associated geochemical data generated in this study provide a foundation for further exploration of microbial resources and environmental adaptation in TP.

## Electronic supplementary material

Additional file 1: Table S1: Field measurement, and soil physical and chemical data of 20 samples in the Tibetan Plateau. **Table S2.** Lithophile and siderophile elements of 20 sampling sites in the Tibetan Plateau. **Table S3.** Proportion of each bacterial phylum in TP samples. **Table S4.** Simpson Diversity Index (1/D), Species Richness and Evenness in TP samples. **Table S5.** P values represent the taxonomic and functional dissimilarities permuted between the upper, lower and upper vs. lower samples of farmland and alpine meadow groups. NS (not significant; P > 0.05), * P < 0.05, ** P < 0.005. (DOC 188 KB)

Additional file 2: Figure S1: Similarity between metagenomic reads and their reference sequences in the public database. Sequencing data were BLASTXed with NR database. Only the reads assigned to the top 20 genera were calculated. A dotted line represents reads assigned to an insect *Acyrthosiphon pisum*. (PDF 141 KB)

Additional file 3: Figure S2: The top abundant 50 bacterial genera in TP soils (L1U - G6U) compared with the abundance of these genera in other 20 environmental samples (WGSHS - BroadIF). The order of these bacterial genera is ranked based on the sum of the bacterial abundance in farmland and meadow samples of TP. “others” represent the remaining genera excluded these 50 genera.; The order of the samples from up to down is ranked based on the proportion of the most predominant genus “*Candidatus Solibacter*” in TP soil and other environmental samples, respectively. (PDF 647 KB)

Additional file 4: Table S6: Samples downloaded from MG-RAST for PCA at phyla level. **Table S7.** Distribution and frequency of reads from G5U aligning to Thiobacillus denitrificans genome. (XLSX 218 KB)

Additional file 5: Figure S3: Distribution of metagenomic reads along the reference genomic sequence of *Thiobacillus denitrificans* (NC_007404.1). The x-axis represents the reference genome (size 2.91 Mb), and the y-axis shows the depth of coverage. (PDF 198 KB)
