# Supplementary material for: Soil bacterial communities shaped by geochemical factors and land use in a less-explored area, Tibetan Plateau
Source: BMC Genomics. 2013 Nov 22;14(1):820. doi: 10.1186/1471-2164-14-820 (PMC4046825; doi:10.1186/1471-2164-14-820)
Supplement: Supplementary file 1 — Additional file 1: Table S1: Field measurement, and soil physical and chemical data of 20 samples in the Tibetan Plateau. Table S2. Lithophile and siderophile elements of 20 sampling sites in the Tibetan Plateau. Table S3. Proportion of each bacterial phylum in TP samples. Table S4. Simpson Diversity Index (1/D), Species Richness and Evenness in TP samples. Table S5. P values represent the taxonomic and functional dissimilarities permuted between the upper, lower and upper vs. lower samples of farmland and alpine meadow groups. NS (not significant; P > 0.05), * P < 0.05, ** P < 0.005. (DOC 188 KB) [file 12864_2013_5515_MOESM1_ESM.doc]

**Table S1 Field measurement, and soil physical and chemical data of 20 sample**s in the Tibetan Plateau.

| **Samples** | **Altitude(km)** | **Temperature (^o^C)** | **Mean grain size (µm)** | **pH** | **TOC%** | **TN%** | **P%** | **S%** | **Moisture (%)** | **C:N** | **C:S** |
| --- | --- | --- | --- | --- | --- | --- | --- | --- | --- | --- | --- |
| **F1U** | 4268 | 28.4 | 5.66 | 8.03 | 2.47 | 0.23 | 1.05 | 0.02 | 8.72 | 10.69 | 107.39 |
| **F1L** | 4268 | 28.4 | 6.02 | 7.86 | 3.19 | 0.19 | 1.41 | 0.02 | 14.34 | 17.09 | 145.00 |
| **F2U** | 3690 | 26.6 | 5.63 | 7.82 | 1.32 | 0.18 | 0.89 | 0.02 | 14.40 | 7.51 | 77.65 |
| **F2L** | 3690 | 26.6 | 5.73 | 7.65 | 1.19 | 0.17 | 0.83 | 0.02 | 15.85 | 7.04 | 74.38 |
| **F3U** | 3693 | 22.0 | 5.68 | 8.09 | 1.34 | 0.18 | 1.07 | 0.03 | 15.55 | 7.53 | 47.86 |
| **F3L** | 3693 | 22.0 | 6.30 | 8.71 | 1.13 | 0.10 | 0.72 | 0.02 | 14.90 | 11.39 | 62.78 |
| **G1U** | 4307 | 20.3 | 4.74 | 7.49 | 2.73 | 0.29 | 0.68 | 0.02 | 22.55 | 9.38 | 118.70 |
| **G1L** | 4307 | 20.3 | 4.97 | 6.08 | 2.09 | 0.15 | 0.78 | 0.02 | 22.38 | 13.78 | 116.11 |
| **G2U** | 4996 | 12.6 | 4.31 | 7.48 | 7.69 | 0.96 | 1.00 | 0.04 | 40.51 | 8.02 | 183.10 |
| **G2L** | 4996 | 12.6 | 4.32 | 7.62 | 8.63 | 0.73 | 0.99 | 0.07 | 9.20 | 11.75 | 128.81 |
| **G3U** | 4996 | 10.4 | 5.13 | 8.48 | 3.67 | 0.08 | 0.94 | 0.07 | 27.69 | 45.14 | 52.43 |
| **G3L** | 4996 | 10.4 | 5.11 | 8.67 | 2.38 | 0.11 | 0.76 | 0.04 | 25.15 | 22.61 | 59.50 |
| **G4U** | 4447 | 15.4 | 4.55 | 7.65 | 7.56 | 0.26 | 0.37 | 0.30 | 29.90 | 28.59 | 25.20 |
| **G4L** | 4447 | 15.4 | 5.63 | 8.15 | 9.49 | 0.32 | 0.39 | 0.73 | 39.27 | 29.29 | 13.00 |
| **G5U** | 4447 | 17.0 | 5.49 | 8.67 | 10.84 | 0.26 | 0.15 | 0.54 | 51.19 | 41.30 | 20.07 |
| **G5L** | 4447 | 17.0 | 5.79 | 8.52 | 10.57 | 0.25 | 0.14 | 0.74 | 50.10 | 41.64 | 14.28 |
| **G6U** | 4295 | 21.5 | 5.31 | 8.65 | 5.38 | 0.24 | 0.88 | 0.04 | 24.85 | 22.31 | 125.12 |
| **G6L** | 4295 | 21.5 | 6.33 | 8.44 | 3.14 | 0.26 | 0.66 | 0.03 | 21.20 | 12.16 | 98.13 |
| **L1U** | 4740 | 16.0 | 1000~500 | 9.35 | 0.76 | 0.01 | 0.09 | 0.02 | ~95.00 | 65.53 | 36.38 |
| **L1L** | 4740 | 16.0 | 2000~1000 | 9.35 | 0.66 | 0.01 | 0.08 | 0.02 | 100.00 | 54.67 | 43.73 |

**Table S2 Lithophile and siderophile elements of 20 sampling sites in the Tibetan Plateau.**

| **Samples** | **Lithophile majors (mg/g)** | | | | | **K:P** | **K:N** | **Siderophile majors (mg/g)** | | **Minors (mg/g)** | | | | | | | | | | |
| --- | --- | --- | --- | --- | --- | --- | --- | --- | --- | --- | --- | --- | --- | --- | --- | --- | --- | --- | --- | --- |
|  | **Al** | **Ca** | **Mg** | **K** | **Na** |  |  | **Fe** | **Mn** | **As** | **Ba** | **Co** | **Cr** | **Cu** | **Li** | **Ni** | **Sr** | **V** | **Zn** | **Ti** |
| **F1U** | 62.67 | 9.61 | 7.37 | 21.42 | 8.29 | 204.4 | 92.7 | 33.46 | 0.59 | 0.07 | 0.28 | 0.02 | 0.07 | 0.01 | 0.04 | 0.03 | 0.14 | 0.07 | 0.09 | 2.93 |
| **F1L** | 55.82 | 12.76 | 6.71 | 21.75 | 6.58 | 154.8 | 116.5 | 31.21 | 0.68 | 0.08 | 0.32 | 0.02 | 0.06 | 0.01 | 0.03 | 0.02 | 0.13 | 0.07 | 0.09 | 3.11 |
| **F2U** | 62.17 | 12.32 | 7.09 | 23.77 | 13.81 | 265.8 | 135.2 | 30.17 | 0.57 | 0.05 | 0.36 | 0.02 | 0.08 | 0.02 | 0.04 | 0.03 | 0.17 | 0.07 | 0.10 | 3.39 |
| **F2L** | 62.31 | 12.36 | 7.12 | 23.94 | 14.14 | 289.6 | 141.6 | 30.28 | 0.55 | 0.04 | 0.36 | 0.02 | 0.10 | 0.01 | 0.04 | 0.03 | 0.18 | 0.07 | 0.08 | 3.34 |
| **F3U** | 63.36 | 17.42 | 7.09 | 23.59 | 14.06 | 221.1 | 132.5 | 29.13 | 0.54 | 0.04 | 0.33 | 0.02 | 0.09 | 0.02 | 0.04 | 0.03 | 0.18 | 0.07 | 0.08 | 3.08 |
| **F3L** | 61.55 | 14.32 | 6.61 | 24.11 | 14.35 | 333.2 | 243.0 | 27.12 | 0.50 | 0.04 | 0.36 | 0.02 | 0.08 | 0.02 | 0.04 | 0.02 | 0.18 | 0.06 | 0.07 | 2.98 |
| **G1U** | 61.04 | 6.19 | 6.10 | 21.34 | 9.21 | 313.7 | 73.3 | 32.45 | 0.68 | 0.06 | 0.35 | 0.02 | 0.08 | 0.01 | 0.04 | 0.03 | 0.12 | 0.07 | 0.09 | 3.53 |
| **G1L** | 61.60 | 5.06 | 6.40 | 21.85 | 8.51 | 278.9 | 144.0 | 38.75 | 0.72 | 0.08 | 0.38 | 0.02 | 0.09 | 0.00 | 0.04 | 0.03 | 0.10 | 0.08 | 0.10 | 3.93 |
| **G2U** | 65.58 | 41.95 | 9.01 | 13.39 | 8.39 | 134.2 | 14.0 | 39.02 | 0.56 | 0.06 | 0.35 | 0.02 | 0.10 | 0.02 | 0.05 | 0.03 | 0.78 | 0.09 | 0.07 | 4.12 |
| **G2L** | 61.80 | 19.50 | 7.31 | 14.5 | 8.06 | 146.7 | 19.7 | 60.64 | 0.47 | 0.07 | 0.41 | 0.03 | 0.11 | 0.03 | 0.04 | 0.04 | 0.32 | 0.09 | 0.09 | 4.14 |
| **G3U** | 60.71 | 89.02 | 9.96 | 12.81 | 6.83 | 136.5 | 157.6 | 46.69 | 0.84 | 0.17 | 0.31 | 0.03 | 0.09 | 0.05 | 0.05 | 0.06 | 0.86 | 0.08 | 0.09 | 4.32 |
| **G3L** | 65.19 | 64.32 | 9.97 | 13.69 | 7.89 | 179.2 | 130.0 | 47.13 | 0.49 | 0.16 | 0.32 | 0.03 | 0.09 | 0.05 | 0.05 | 0.06 | 0.78 | 0.08 | 0.09 | 4.58 |
| **G4U** | 34.57 | 183.80 | 4.55 | 9.891 | 6.88 | 268.9 | 37.4 | 17.53 | 0.24 | 0.05 | 0.24 | 0.01 | 0.04 | 0.01 | 0.03 | 0.02 | 1.12 | 0.04 | 0.04 | 2.36 |
| **G4L** | 39.64 | 193.90 | 6.28 | 11.15 | 6.85 | 287.4 | 34.4 | 20.82 | 0.27 | 0.05 | 0.27 | 0.01 | 0.04 | 0.01 | 0.04 | 0.02 | 1.22 | 0.06 | 0.04 | 2.08 |
| **G5U** | 26.12 | 269.60 | 3.81 | 6.763 | 2.82 | 456.7 | 25.8 | 12.73 | 0.18 | 0.03 | 0.16 | 0.01 | 0.03 | 0.02 | 0.02 | 0.01 | 0.65 | 0.04 | 0.02 | 1.33 |
| **G5L** | 24.44 | 273.80 | 3.24 | 6.323 | 2.69 | 448.1 | 24.9 | 12.35 | 0.15 | 0.05 | 0.15 | 0.01 | 0.03 | 0.01 | 0.02 | 0.02 | 0.62 | 0.04 | 0.02 | 1.24 |
| **G6U** | 54.38 | 26.46 | 6.75 | 23.54 | 6.99 | 267.2 | 97.6 | 25.52 | 0.46 | 0.05 | 0.36 | 0.02 | 0.09 | 0.01 | 0.04 | 0.03 | 0.14 | 0.07 | 0.07 | 3.23 |
| **G6L** | 59.87 | 18.09 | 6.85 | 25.14 | 8.29 | 381.4 | 97.4 | 30.88 | 0.33 | 0.05 | 0.36 | 0.02 | 0.11 | 0.02 | 0.03 | 0.03 | 0.12 | 0.07 | 0.08 | 3.96 |
| **L1U** | 23.40 | 174.10 | 34.02 | 16.75 | 6.13 | 1824.4 | 1431.6 | 3.94 | 0.23 | 0.06 | 0.25 | 0.00 | 0.01 | 0.00 | 0.01 | 0.01 | 0.28 | 0.01 | 0.01 | 0.38 |
| **L1L** | 19.26 | 163.70 | 31.75 | 9.83 | 5.56 | 1175.4 | 819.2 | 6.81 | 0.31 | 0.04 | 0.16 | 0.01 | 0.01 | 0.00 | 0.01 | 0.00 | 0.22 | 0.02 | 0.02 | 1.20 |

**Table S3 Proportion of each bacterial phylum in TP samples.**

| **Phylum (%)** | F1U | F1L | | F2U | | F2L | | F3U | | F3L | | G1U | | G1L | | G2U | | G2L | | G3U | | G3L | | G4U | | G4L | | G5U | | G5L | | G6U | | G6L | | L1U | | L1L | |
| --- | --- | --- | --- | --- | --- | --- | --- | --- | --- | --- | --- | --- | --- | --- | --- | --- | --- | --- | --- | --- | --- | --- | --- | --- | --- | --- | --- | --- | --- | --- | --- | --- | --- | --- | --- | --- | --- | --- | --- |
| **Proteobacteria** | 56.4 | 57.7 | 43.7 | | 44.3 | | 56.3 | | 55.6 | | 51.1 | | 45.0 | | 49.6 | | 49.2 | | 66.2 | | 56.9 | | 62.0 | | 71.2 | | 69.1 | | 54.5 | | 41.4 | | 53.5 | | 53.5 | | 44.5 | |  |
| **Actinobacteria** | 10.7 | 12.0 | 5.6 | | 6.0 | | 12.7 | | 10.0 | | 14.3 | | 13.6 | | 6.8 | | 4.7 | | 9.3 | | 15.5 | | 13.0 | | 6.2 | | 4.4 | | 2.2 | | 17.9 | | 13.9 | | 5.1 | | 6.4 | |  |
| **Bacteroidetes** | 7.1 | 3.8 | 15.3 | | 12.1 | | 5.3 | | 3.6 | | 5.0 | | 2.0 | | 9.6 | | 6.5 | | 3.8 | | 3.6 | | 4.8 | | 3.5 | | 7.4 | | 28.6 | | 2.7 | | 2.9 | | 20.4 | | 17.8 | |  |
| **Acidobacteria** | 7.3 | 9.0 | 10.8 | | 11.7 | | 7.2 | | 9.5 | | 8.6 | | 16.3 | | 10.3 | | 14.2 | | 4.5 | | 6.0 | | 3.6 | | 3.3 | | 1.9 | | 1.0 | | 14.7 | | 9.7 | | 2.0 | | 2.8 | |  |
| **Verrucomicrobia** | 4.9 | 4.4 | 9.0 | | 8.6 | | 4.3 | | 3.8 | | 6.4 | | 7.0 | | 8.1 | | 7.3 | | 5.1 | | 3.7 | | 4.2 | | 3.2 | | 4.5 | | 1.8 | | 10.4 | | 4.2 | | 3.9 | | 7.0 | |  |
| **Planctomycetes** | 3.5 | 3.0 | 3.3 | | 3.9 | | 3.8 | | 4.0 | | 3.6 | | 2.4 | | 3.4 | | 3.6 | | 2.6 | | 4.4 | | 2.9 | | 2.0 | | 2.2 | | 1.2 | | 3.8 | | 3.5 | | 6.9 | | 11.3 | |  |
| **Chloroflexi** | 1.6 | 1.7 | 2.5 | | 3.6 | | 2.3 | | 3.4 | | 2.6 | | 3.1 | | 3.0 | | 3.3 | | 2.6 | | 2.3 | | 2.1 | | 2.7 | | 2.6 | | 2.8 | | 3.0 | | 2.9 | | 0.9 | | 1.6 | |  |
| **Firmicutes** | 1.8 | 1.9 | 2.5 | | 2.6 | | 2.0 | | 2.6 | | 2.3 | | 3.2 | | 2.7 | | 3.0 | | 2.0 | | 1.8 | | 1.9 | | 2.2 | | 2.4 | | 4.0 | | 2.1 | | 2.7 | | 1.5 | | 1.8 | |  |
| **Gemmatimonadetes** | 3.4 | 3.0 | 2.8 | | 2.2 | | 2.5 | | 2.8 | | 3.0 | | 2.2 | | 1.4 | | 1.6 | | 1.3 | | 2.4 | | 3.0 | | 2.8 | | 3.1 | | 0.7 | | 1.0 | | 3.4 | | 2.1 | | 1.8 | |  |
| **Cyanobacteria** | 1.6 | 1.3 | 1.9 | | 2.0 | | 1.4 | | 1.8 | | 1.6 | | 1.7 | | 2.2 | | 3.1 | | 1.1 | | 1.4 | | 1.1 | | 1.0 | | 0.8 | | 0.7 | | 1.7 | | 1.5 | | 1.1 | | 1.4 | |  |
| **Nitrospirae** | 0.6 | 1.1 | 0.9 | | 1.6 | | 1.0 | | 1.5 | | 0.4 | | 2.0 | | 1.4 | | 1.8 | | 0.4 | | 1.0 | | 0.2 | | 0.4 | | 0.2 | | 0.2 | | 0.2 | | 0.6 | | 1.5 | | 2.3 | |  |

**Table S4 Simpson Diversity Index (1/D), Species Richness and Evenness in TP samples.**

|  | **F1U** | **F1L** | **F2U** | **F2L** | **F3U** | **F3L** | **G1U** | **G1L** | **G2U** | **G2L** | **G3U** | **G3L** | **G4U** | **G4L** | **G5U** | **G5L** | **G6U** | **G6L** | **L1U** | **L1L** |
| --- | --- | --- | --- | --- | --- | --- | --- | --- | --- | --- | --- | --- | --- | --- | --- | --- | --- | --- | --- | --- |
| **Simpson Diversity Index**  **(1/D)** | 51.90 | 49.94 | 47.50 | 45.82 | 58.94 | 50.34 | 48.07 | 35.37 | 58.23 | 43.87 | 68.75 | 66.79 | 53.60 | 24.40 | 70.81 | 58.28 | 32.69 | 46.86 | 30.91 | 22.37 |
| **Evenness** | 0.76 | 0.75 | 0.76 | 0.76 | 0.76 | 0.76 | 0.74 | 0.71 | 0.77 | 0.76 | 0.78 | 0.77 | 0.77 | 0.73 | 0.81 | 0.77 | 0.69 | 0.75 | 0.74 | 0.76 |
| **Species Richness** | 756 | 747 | 673 | 723 | 761 | 752 | 762 | 766 | 722 | 639 | 760 | 748 | 769 | 772 | 517 | 705 | 756 | 761 | 783 | 537 |

# Table S5 P values represent the taxonomic and functional dissimilarities permuted between the upper, lower and upper vs. lower samples of farmland and alpine meadow groups. NS (not significant; P>0.05), * P<0.05, ** P<0.005.

| **Taxonomy** | **Farmland upper** | **Farmland lower** | **Farmland upper-lower** | **Alpine meadow upper** | **Alpine meadow upper-lower** |
| --- | --- | --- | --- | --- | --- |
| **Farmland lower** | NS |  |  |  |  |
| **Farmland upper-lower** | * | * |  |  |  |
| **Alpine meadow upper** | * | * | ** |  |  |
| **Alpine meadow lower** | * | * | ** | NS |  |
| **Alpine meadow upper-lower** | * | * | * | * | * |

| **Function** | **Farmland upper** | **Farmland lower** | **Farmland upper-lower** | **Alpine meadow upper** | **Alpine meadow upper-lower** |
| --- | --- | --- | --- | --- | --- |
| **Farmland lower** | * |  |  |  |  |
| **Farmland upper-lower** | * | * | * |  |  |
| **Alpine meadow upper** | NS | * | * |  |  |
| **Alpine meadow lower** | * | * | * | NS |  |
| **Alpine meadow upper-lower** | NS | NS | * | * | * |
