# Supplementary figures and images for: Soil bacterial communities shaped by geochemical factors and land use in a less-explored area, Tibetan Plateau
Source: BMC Genomics. 2013 Nov 22;14(1):820. doi: 10.1186/1471-2164-14-820 (PMC4046825; doi:10.1186/1471-2164-14-820)

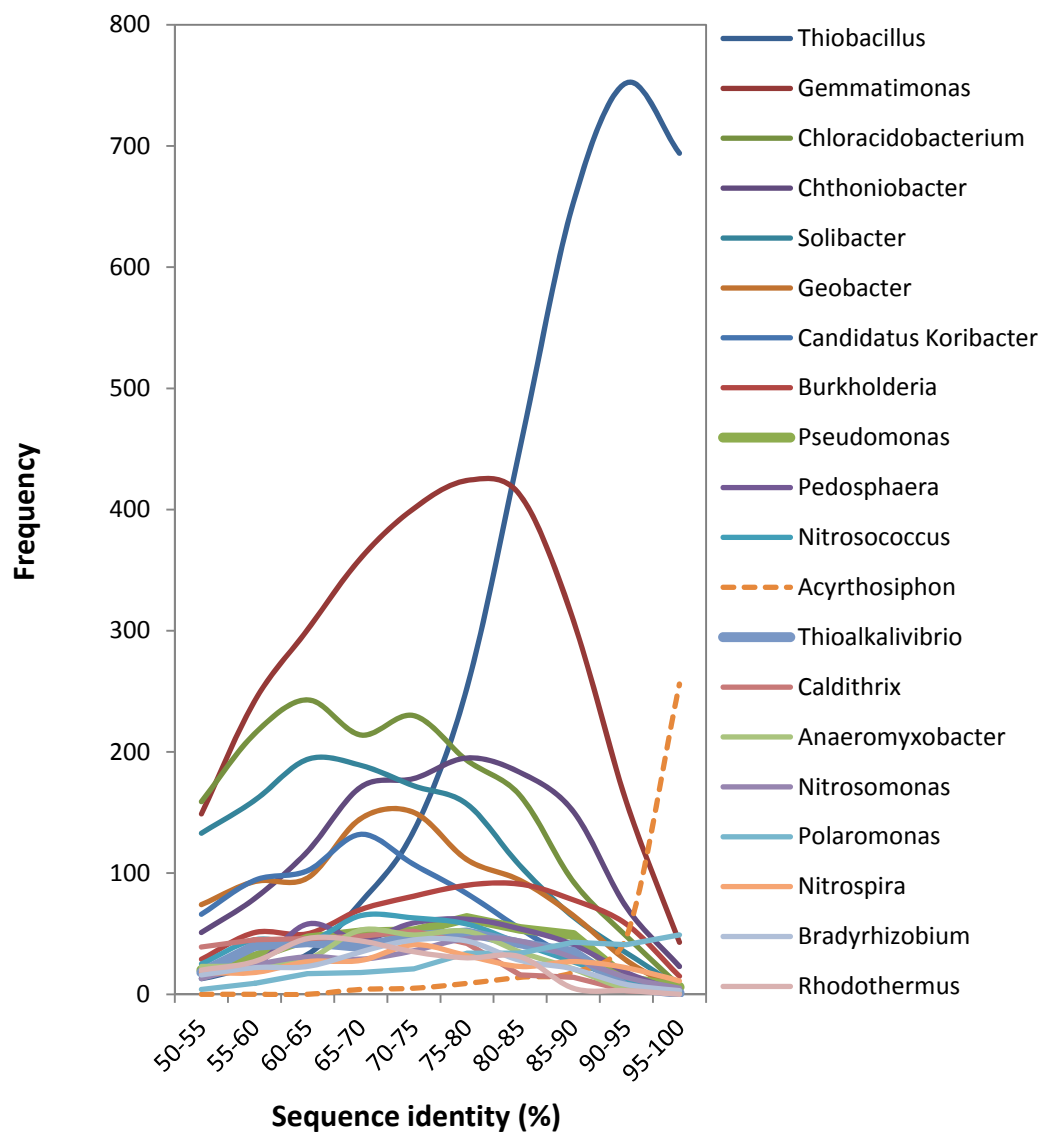

Supplement: Supplementary file 2 — Additional file 2: Figure S1: Similarity between metagenomic reads and their reference sequences in the public database. Sequencing data were BLASTXed with NR database. Only the reads assigned to the top 20 genera were calculated. A dotted line represents reads assigned to an insect Acyrthosiphon pisum. (PDF 141 KB) [file 12864_2013_5515_MOESM2_ESM.pdf]

Relative Abundance (%)

0% 10% 20% 30% 40% 50% 60% 70% 80% 90% 100%

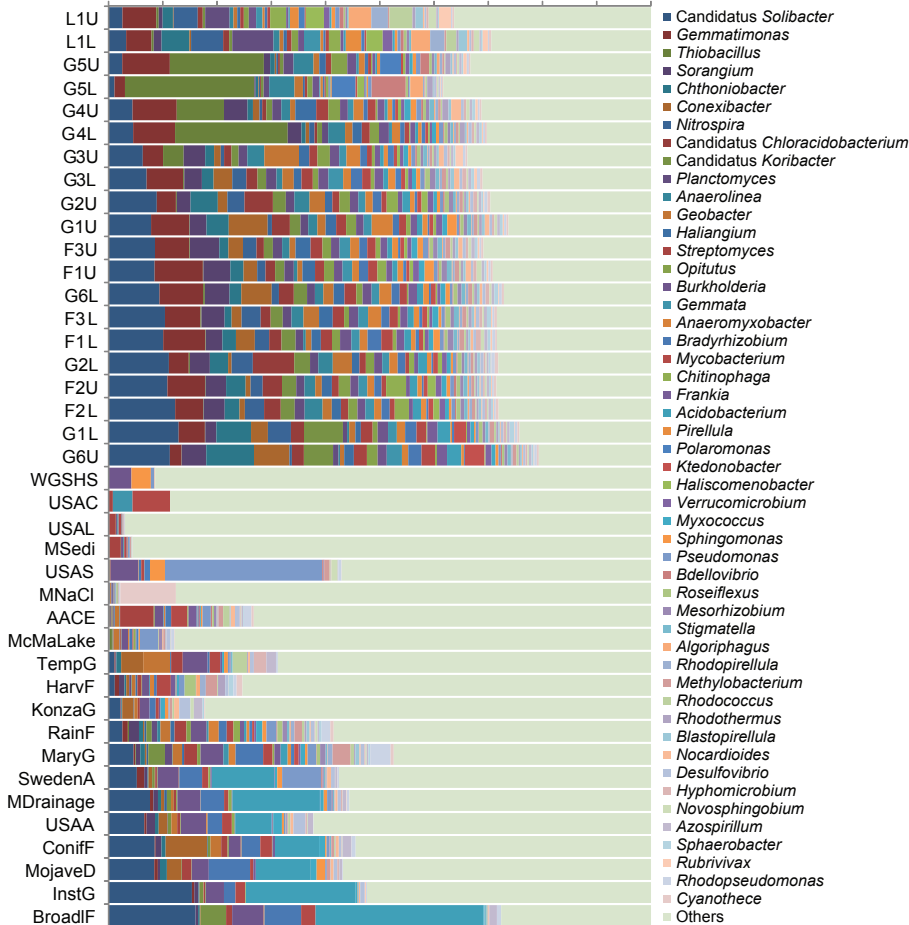

Supplement: Supplementary file 3 — Additional file 3: Figure S2: The top abundant 50 bacterial genera in TP soils (L1U - G6U) compared with the abundance of these genera in other 20 environmental samples (WGSHS - BroadIF). The order of these bacterial genera is ranked based on the sum of the bacterial abundance in farmland and meadow samples of TP. “others” represent the remaining genera excluded these 50 genera.; The order of the samples from up to down is ranked based on the proportion of the most predominant genus “Candidatus Solibacter” in TP soil and other environmental samples, respectively. (PDF 647 KB) [file 12864_2013_5515_MOESM3_ESM.pdf]

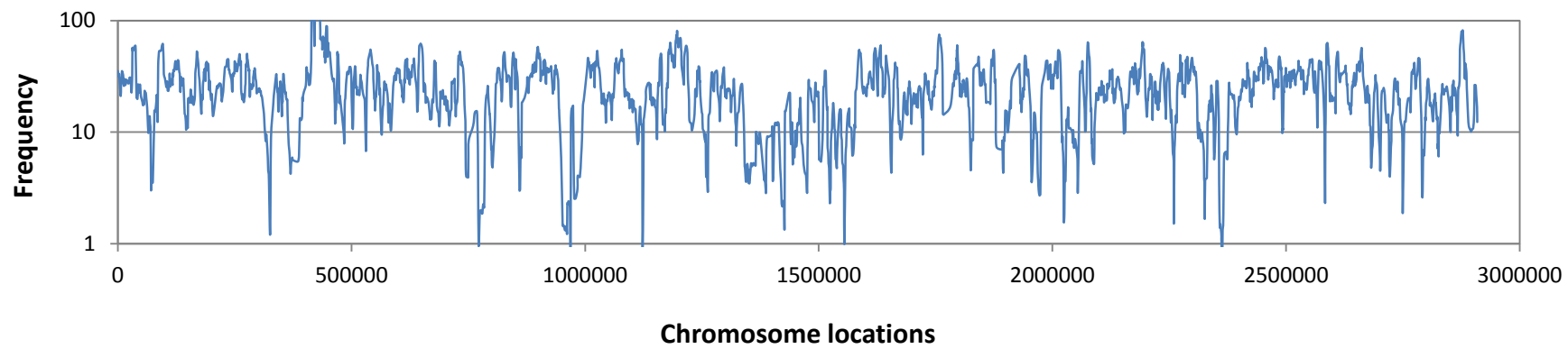

Supplement: Supplementary file 5 — Additional file 5: Figure S3: Distribution of metagenomic reads along the reference genomic sequence of Thiobacillus denitrificans (NC_007404.1). The x-axis represents the reference genome (size 2.91 Mb), and the y-axis shows the depth of coverage. (PDF 198 KB) [file 12864_2013_5515_MOESM5_ESM.pdf]
